# Do changes in body mass alter white blood cell profiles and immune function in Australian cane toads (*Rhinella marina*)?

**DOI:** 10.1098/rstb.2022.0122

**Published:** 2023-07-31

**Authors:** Gregory P. Brown, Cameron M. Hudson, Richard Shine

**Affiliations:** ^1^ School of Natural Sciences, Macquarie University, Sydney, New South Wales 2109, Australia; ^2^ Department of Fish Ecology and Evolution, EAWAG Swiss Federal Institute of Aquatic Science and Technology, Center of Ecology, Evolution, and Biochemistry, Seestrasse 79, CH-6047 Kastanienbaum, Switzerland; ^3^ Department of Aquatic Ecology, Eawag, Swiss Federal Institute of Aquatic Science and Technology, Ueberlandstrasse 133, CH-8600 Dübendorf, Zürich, Switzerland

**Keywords:** *Bufo marinus*, ecoimmunology, stress, energy budget, leukogram

## Abstract

Variation in food resources can result in dramatic fluctuations in the body condition of animals dependent on those resources. Decreases in body mass can disrupt patterns of energy allocation and impose stress, thereby altering immune function. In this study, we investigated links between changes in body mass of captive cane toads (*Rhinella marina*), their circulating white blood cell populations, and their performance in immune assays. Captive toads that lost weight over a three-month period had increased levels of monocytes and heterophils and reduced levels of eosinophils. Basophil and lymphocyte levels were unrelated to changes in mass. Because individuals that lost mass had higher heterophil levels but stable lymphocyte levels, the ratio of these cell types was also higher, partially consistent with a stress response. Phagocytic ability of whole blood was higher in toads that lost mass, owing to increased circulating levels of phagocytic cells. Other measures of immune performance were unrelated to mass change. These results highlight the challenges faced by invasive species as they expand their range into novel environments which may impose substantial seasonal changes in food availability that were not present in the native range. Individuals facing energy restrictions may shift their immune function towards more economical and general avenues of combating pathogens.

This article is part of the theme issue ‘Amphibian immunity: stress, disease and ecoimmunology’.

## 1. Introduction

The availability and/or quality of food is rarely constant over time for any organism, with substantial variation induced by factors such as seasonal variation in environmental conditions or the abundance of prey [1–5]. Reflecting that variation, individuals of many taxa exhibit considerable fluctuations in body mass through time, with decreases during periods of resource limitation and fattening during periods of resource abundance. Given the near-ubiquity of fluctuations in body condition through time, we expect local adaptation and/or phenotypic plasticity to have fine-tuned responses to that situation. For example, animals that encounter low food availability may respond by reducing their rate of metabolic expenditure via reducing activity levels or selecting lower body temperatures (in ectotherms: [6]) and by going into torpor (in endotherms: [7]). In some species, loss of body mass may be an adaptive tactic to reduce locomotor or maintenance costs [8].

The magnitude of mass loss varies substantially among species, driven by issues such as the stochasticity and seasonality of food availability. Many of the most spectacular cases occur among ectothermic animals, perhaps because large fat bodies compromise rates of thermal exchange across the body surface in endotherms [9]. By contrast, an ectotherm can store large amounts of energy without affecting its thermal biology [9]. High storage capacity provides an opportunity for the organism to accumulate energy during brief periods of resource abundance, and then slowly expend that energy for maintenance over the following months or years (e.g. [10]). Large fluctuations in body mass may also be common among migratory or invasive species, because of the high energy demands of sustained dispersal [11]. Body mass fluctuations in invasive species may also depend how close the population is to the invasion front. Processes such as spatial sorting for enhanced dispersal among frontal populations may alter energy demands and associated trade-offs [12]. The effects of unpredictable resource availability, and temporal variation in rates of feeding, may be especially pronounced in invasive species if they did not evolve under these conditions but encounter them in the invaded range [3].

In addition, the spatio-temporal shifts in population density that occur during range expansion influence competition for resources among conspecifics and thus, energy intake [13–15]. Temporal fluctuations in feeding rates and body condition may be especially likely as a species invades increasingly seasonal or stochastically variable environments.

Regardless of the cause, variation in body mass inevitably affects energy-allocation decisions [16]. Patterns of investment into competing compartments—growth, storage, reproduction, maintenance etc.—must be altered during periods when energy intake is low. The immune system is one such component, likely to be reconfigured when energy is scarce [17,18]. Immunocompetence is a strong target of selection because pathogens can adversely affect the host's health and fitness. However, mounting a robust immune response can require substantial energy [19,20]. In addition to the cost of mounting a full-scale immune reaction, manufacturing and maintaining the cells and substances required for robust immune surveillance can be high [21]. An individual's immune configuration is therefore likely to be tightly linked to the amount of energy it has available, in light of competing demands such as growth and reproduction [19]. Energetically expensive immune components may need to be sacrificed and sparse resources redirected in favour of less expensive mechanisms [19]. One way to fine-tune investment is to allocate resources differentially to components of the immune system that provide general and relatively low-cost defence versus components that can protect more effectively against specific challenges but are costly to induce [22–24].

Another means by which reduced energy intake could affect immune function is if it elicits a stress response and triggers the release of glucocorticoids such as corticosterone (CORT) [25,26]. Although the links between CORT and stress are complex [27,28], CORT plays a role in metabolism, mobilization and redirection of energy stores, and levels often increase during periods of food deprivation [25]. Another effect of glucocorticoids is alteration of the immune system. For example, increased CORT levels reconfigure the population of circulating white blood cells (WBCs). Lymphocytes migrate out of circulation and are sequestered in the bloodstream and into tissues, while heterophils are recruited into circulation and retained there. As a result of this redistribution of different cell types into and out of circulation, the ratio of heterophils to lymphocytes in the blood can be used as an index of circulating CORT concentration, and hence stress levels [29]. CORT can also modulate immune responses by altering the distribution and activity of cytokines and other immune agents [30–32]

The abundance, wide geographical distribution, and large body size of cane toads (*Rhinella marina*) have encouraged researchers to use these toads as a model species for examining physiological issues, including ecoimmunology. The aim of the present study was to determine whether the immune defences of cane toads are correlated with their energy balance. We quantified changes in body mass in captive-reared cane toads and measured immune parameters at the time of their final weighing.

If the production and maintenance of immune products is constrained by energy balance, we predicted that toads in positive energy balance (i.e. individuals that gained mass over the study period) would exhibit higher performance in standardized immune assays than would conspecifics in poorer energy balance (i.e. that lost mass over the same period). However, if mass loss imposed stress, thereby increasing circulating heterophil concentration, immune functions performed by heterophils (i.e. phagocytosis) might be enhanced among individuals that lost weight.

## 2. Material and methods

### (a) Study species

Native to South America, cane toads (*Rhinella marina*, formerly *Bufo marinus*) have been translocated to many countries for control of insect pests [33]. The climate in the toads' native range and in serial translocation sites (Caribbean and Hawai'i) prior to their translocation to Australia is wet-tropical, with rain falling during every month of the year [3]. Toads were released in Queensland on Australia's east coast in 1935 and have spread more than 2000 km westwards since then, experiencing progressively more dramatic seasonality in rainfall (including sequences of rain-free months each year) as they advanced [3]. Our study was carried out at a field station 60 km east of the city of Darwin on the Adelaide River floodplain (12.579° S, 131.314° E) in the Northern Territory. The area experiences a wet–dry tropical climate, with maximum daily air temperatures greater than 32°C year-round. Precipitation is highly seasonal, with rainfall largely restricted to the November–April wet season each year [34].

### (b) Mass change and immune measures in captive cane toads

Our studies at the field station 60 km east of the city of Darwin included captive breeding of parental toads from sites across the Australian range and raising the progeny to adulthood under standardized ‘common garden' conditions. The advantages of using captive-reared toads to study immune performance is that factors such as relatedness and age are known and prior exposure to pathogens and parasites can be minimized. Offspring from captive breedings were reared in large (110 × 110 × 70 cm) open-topped outdoor enclosures and used in studies to evaluate heritability of various traits (for further details on common garden rearing see [35]). All enclosures provided access to water and shade, and food was provided by artificial lights hanging above each enclosure, which attracted nocturnal insects. Commercially purchased cockroaches and mealworms dusted with vitamin powder were occasionally added to each enclosure as supplemental food. Nocturnal insects are present year-round, but especially abundant during the wet season, when this study took place. At the time of this study, the number of toads in each enclosure ranged from 6 to 17, grouped by similar body sizes.

We periodically recaptured toads from the enclosures to measure them and/or conduct performance assays [36–38]. The present paper is based on data from 154 adult toads that had been bred in captivity and reared all their lives in outdoor enclosures. The toads were weighed during the wet season in January 2016, several months after they had last undergone any performance trials. Then, two to three months later (when the toads were 17–24 months old), they were weighed a second time. The difference in mass between the first and second weighing was divided by the number of days elapsed to calculate the rate of weight change. Immediately after their second weighing toads underwent a series of immunological assays (see below).

We obtained a 0.2 ml blood sample from each toad via cardiocentesis using a heparinized syringe. To minimize the effects of handling time on blood components used for immune assays, samples were obtained within 3 min of a toad being removed from its enclosure [28]. The toad was then weighed and its snout–urostyle length (SUL, mm) was measured. For immune measures (see below), we used 40 µl of whole blood to perform a phagocytosis assay, 5 µl for haemocytometry and 5 µl to prepare a blood smear. The remaining blood sample was centrifuged for 4 min at 13 000*g* to provide plasma for a bactericidal assay.

We performed three challenge-based immune assays, whereby immune function is stimulated by the introduction of a pathogen signal. A bacteria-killing assay and phagocytosis chemiluminescence assay assessed components of the innate immune system, which is fast-acting and non-specific. A phytohaemagglutinin (PHA) skin-swelling assay assessed the proliferation of lymphocytes at a site where a T-cell mitogen (PHA) was injected [39]. The PHA test is a commonly used assay of cell-mediated immune responses. As a component of the acquired arm of the immune system, such responses are slower to initiate than innate responses but can target specific pathogens [39].

#### (i) Phytohaemagglutinin skin-swelling assay

Immediately after each toad was blood-sampled and weighed, we measured the thickness of the webbing between the second and third toes of each hind foot three times in succession using a dial thickness gauge (Peacock G1-A; Ozaki Manufacturing, Tokyo, Japan). We then injected 0.05 ml of a PHA (L7854, Sigma) solution (2 mg ml^−1^ in sterile phosphate-buffered saline, PBS) into the webbing between the second and third toes of the right hind foot using a 0.3 ml syringe with a 30 G needle. As a procedural control, to measure swelling caused by needle-damage alone, we injected 0.05 ml of sterile PBS into the webbing of the left hind foot.

Toads were then placed into individual damp cotton bags inside a plastic container and held indoors in an air-conditioned room. After 24 h, we removed toads from their cloth bag and the thickness of both injected hind toe-webs was remeasured to quantify the amount of swelling. Toads were then returned to their outdoor enclosures. We calculated the percentage swelling caused by injection of both PBS and PHA as: 100×((24 h thickness − initial thickness)/initial thickness). We also calculated the relative difference by subtracting the percentage swelling (%swelling) caused by PBS from the %swelling caused by PHA.

#### (ii) Phagocytosis assay

This assay measures tested the capacity of phagocytes (mainly heterophils) in toad whole blood to engulf zymosan particles. We added 40 µl of whole blood to 760 µl of sterile amphibian Ringers to produce a 1 : 20 dilution. We then added 240 µl of the diluted blood to duplicate wells of a 96-well microassay plate along with 30 µl of luminol (A8511, Sigma) solution and 10 µl of zymosan (Z4250, Sigma) solution. A control well for each sample contained 240 µl of diluted blood, and 30 µl luminol but with 10 µl of PBS instead of zymosan. Immediately upon addition of zymosan to the wells, the plate was placed in a luminometer (FluoroStar Optima; BMG Labtech, Ortenberg, Germany) and luminescence was read at 5 min intervals over 180 min. Higher luminescence values indicate higher rates of phagocytosis occurring in the sample.

#### (iii) Bacteria killing ability assay

We tested the ability of toad plasma to kill *Escherichia coli* using a modification of Matson *et al*.'s method [40]. A lyophilized pellet of *E. coli* (ATCC 8739) was dissolved in sterile PBS, such that 10 µl contained approximately 900 colony-forming units. Toad plasma was diluted 1 : 10 in sterile CO_2_-independent medium enriched with l-glutamine. We added 10 µl of the diluted *E. coli* solution with 140 µl of diluted plasma from each toad and immediately spread 50 µl of the solution onto a tryptic soy agar plate. The remaining plasma–bacteria mixture was held at 25°C for 60 min and then another 50 µl sample was spread onto an agar plate. To measure bacterial growth in the absence of toad plasma, we also prepared triplicate control samples, where 10 µl of dilute *E. coli* solution was added to 140 µl of sterile CO_2_-independent medium. As with the toad samples, 50 µl aliquots of control samples were plated out at 0 and 60 min. All plates were then incubated at 37°C for 24 h and the number of colonies on each plate was counted. To quantify the bactericidal ability of toad plasma, we used the method of [41], which indexes the 24 h percentage changes in counts of bacteria incubated with toad plasma against 24 h percentage changes in control samples.

#### (iv) Haemocytometry and white blood cell differentials

**Figure 1.**
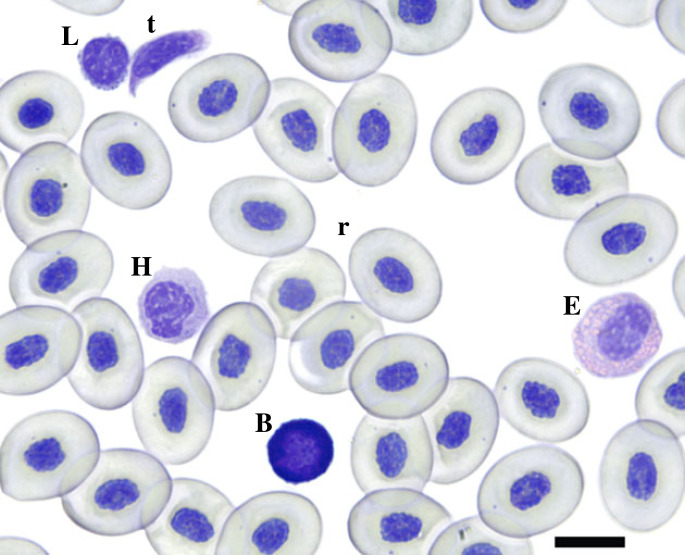
Representative cane toad (*Rhinella marina*) blood cell types. Basophil (B), eosinophil (E), heterophil (H), lymphocyte (L), thrombocyte (t) and erythrocyte (r). Bar represents 10 µm. Giemsa stain. (Online version in colour.)

We used haemocytometry to determine the concentration of blood cells in circulation and used blood smears to make differential counts of WBCs. Blood smears were air-dried overnight, fixed in methanol and stained using modified Giemsa solution. Slides were fitted with a cover glass and examined at 1000× magnification. The first 100 WBCs encountered were identified as either basophil, eosinophil, monocyte, heterophil or lymphocyte (figure 1). For haemocytometry, 5 µl of whole blood was added to 995 ml of Natt–Herrick solution. The solution was refrigerated for 60 min and then vortex-mixed to resuspend the cells. A 10 µl aliquot was then placed onto a haemocytometer and examined in the chamber under 100× magnification to count total red blood cells (RBCs) and total WBCs [42]. Using the differential white cell counts taken from blood smears, the volume of the haemocytometer and the dilution factor (1 : 200) of blood used for haemocytometry, we calculated the concentration of each type of white blood cell per millilitre of whole blood [42].

### (c) Statistical analysis

We used mixed model analyses to assess links between seven measures of immune performance (PBS %swelling, PHA %swelling, PHA–PBS %swelling, mean luminescence, maximum luminescence, time to maximum luminescence, Allen bacteria killing ability (BKA) index) and the rate of mass change of each toad. Sex and body size (SUL) were included as covariates in all models. Litter ID, enclosure ID and assay date were included as random effects to incorporate levels of similarity among siblings, cage mates and assay groups. For graphical purposes, we calculated percentage change in body mass by dividing mass change by initial mass and multiplying by 100. However, in analyses, rate of mass change (g d^−1^) was corrected for initial body size by including SUL as a covariate in the models (see below).

We used the same mixed model structure to assess the relationships between mass change and concentrations of different blood cell types and H : L ratio. We log-transformed concentrations of the five white blood cell types and H : L ratio prior to analyses to satisfy normality and variance assumptions.

To establish which types of WBCs most affected phagocytic performance, we used a model that included the concentration of the cell types as independent variables and mean luminescence (from the phagocytosis assay) as the dependent variable. Clutch ID, enclosure ID and assay date were included as random effects. All mixed model analyses were carried out using Proc Mixed in SAS 9.4 (SAS Institute, Cary, NC, USA).

We used residuals from a regression of log-transformed body mass on log-transformed body length (SUL) to index body condition (mass corrected for body size). To determine if body condition was related to the degree of mass change over the preceding period, we calculated a Pearson correlation between the two variables.

## 3. Results

### (a) Changes in mass of captive toads

Changes in mass of the 154 captive toads re-weighed after 63–107 days ranged from a loss of 36 g to a gain of 78 g (−0.5 to +2.0% of initial mass per day). Fifty-six toads lost weight and 98 gained weight. At the time of second measure, when the immune assays performed, toads that had gained more mass were in better body condition (*r* = 0.45, *p* < 0.0001).

### (b) Relationships between mass change and immune measures

**Figure 2.**
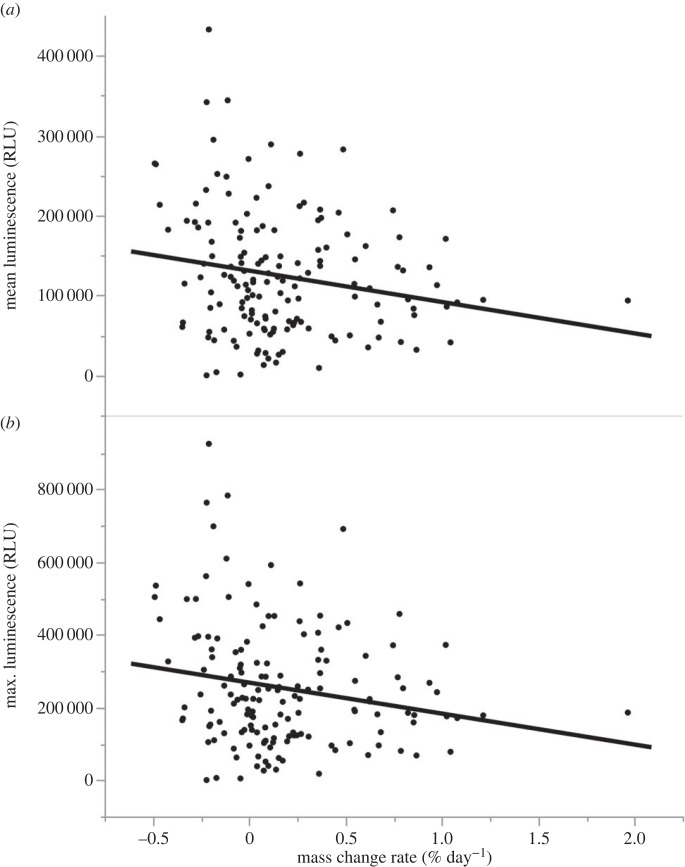
Relationship between rate of mass change (% day^−1^) and phagocytic ability of whole blood. (*a*) Mean luminescence over 180 min and (*b*) maximum luminescence recorded over 180 min. *y*-axis scale is in relative luminescence units (RLU).

None of our challenge-induced immune measures was significantly related to sex or body size (SUL) (all *p* > 0.10, table 1). Immune responses related to toe-web swelling and BKA were also unrelated to the change in mass of toads over three months prior to the assays (all *p* > 0.05, table 1). However, change in mass negatively affected phagocytosis activity, causing a decrease in both average luminescence (estimate = −54292, *F*_1,104_ = 4.78, *p* = 0.031; table 1 and figure 2) and maximum luminescence (estimate = −128721, *F*_1,104_ = 5.41, *p* = 0.022; table 1 and figure 2). Toads that lost mass had higher phagocytic ability than did toads that gained mass.

**Table 1.** Mixed model analyses of the relationships between sex, body size (snout–urostyle length, SUL, mm) and recent change in body mass (% day^−1^) and immune assay performance of captive-reared cane toads (*Rhinella marina*). See text for assay descriptions. Family ID, enclosure number and assay date were included as random effects in all models. Italicized values indicate *p* < 0.05.

	PBS toe-web %swelling		PBS toe-web %swelling		PBS-PHA toe–web %swelling		mean luminescence		maximum luminescence		time to max. luminescence		Allen BKA	
	*F*_1,104_	*p*	*F*_1,104_	*p*	*F*_1,104_	*p*	*F*_1,104_	*p*	*F*_1,104_	*p*	*F*_1,104_	*p*	*F*_1,104_	*p*
sex	1.24	0.2683	0.66	0.4201	1.85	0.1763	0.06	0.8085	0.58	0.4495	2.84	0.0951	1.06	0.3053
body size	0.59	0.4425	0.94	0.3352	2.60	0.1099	1.13	0.2908	1.30	0.2565	2.15	0.1460	2.67	0.1054
mass change rate	0.39	0.5317	0.60	0.4385	0.30	0.5863	*4.78*	*0.0310*	*5.41*	*0.0220*	1.02	0.3142	1.06	0.3062

**Figure 3.**
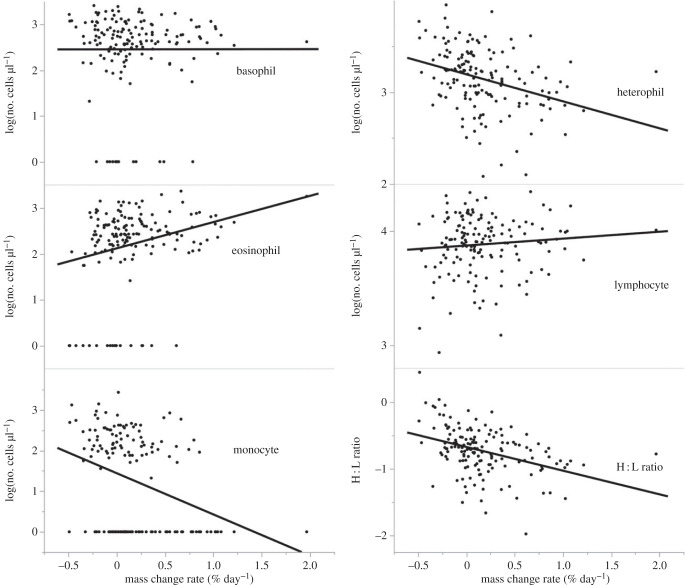
Relationship between rate of mass change (% day^−1^) and log-transformed white blood cell concentrations and heterophil : lymphocyte (H : L) ratios.

Haematological parameters did not differ significantly between male and female toads (all *p* > 0.07, table 2). Body size was positively related to both erythrocyte concentration and eosinophil concentration (both *p* < 0.037, table 2). Change in mass affected the concentration of three of the five types of WBC (figure 3 and table 2). Toads that gained mass prior to the second census had higher concentrations of eosinophils in circulation (estimate = 0.585, *F*_1,104_ = 4.88, *p* = 0.029), but lower levels of monocytes (estimate = −1.35, *F*_1,104_ = 12.85, *p* = 0.0005) and heterophils (estimate = −0.47, *F*_1,104_ = 21.07, *p* < 0.0001). Levels of basophils and lymphocytes were unaffected by changes in mass (figure 3 and table 2). As a result of heterophils decreasing with weight change while lymphocytes levels remained stable, the ratio of heterophils to lymphocytes was negatively related to mass change (estimate = −0.59, *F*_1,104_ = 29.96, *p* < 0.0001; figure 3 and table 2). Toads that had lost mass prior to assays had more heterophils relative to lymphocytes than did toads that had gained mass.

**Table 2.** Mixed model analyses of the relationships between sex, body size (snout–urostyle length, SUL, mm) and recent change in body mass (% day^−1^) and circulating blood cell concentrations (cells ml^−1^) of captive-reared cane toads (*Rhinella marina*). See text for assay descriptions. Family ID, enclosure number and assay date were included as random effects in all models. Italicized values indicate *p* < 0.05.

	RBC concentration		total WBC concentration		basophil concentration		eosinophil concentration		monocyte concentration		lymphocyte concentration		heterophil concentration		H : L ratio	
	*F*_1,104_	*p*	*F*_1,104_	*p*	*F*_1,104_	*p*	*F*_1,104_	*p*	*F*_1,104_	*p*	*F*_1,104_	*p*	*F*_1,104_	*p*	*F*_1,104_	*p*
sex	2.20	0.1411	1.52	0.2208	0.03	0.8645	0.39	0.5325	0.84	0.3627	2.48	0.1186	0.14	0.7138	3.22	0.0757
body size	*4.48*	*0.0367*	0.01	0.9152	0.41	0.5249	*4.83*	*0.0303*	2.78	0.0986	0.08	0.7826	0.58	0.4464	2.15	0.1452
mass change rate	1.43	0.2348	0.13	0.7193	0.48	0.4909	*4.88*	*0.0294*	*12.85*	*0.0005*	1.47	0.2274	*21.07*	*<0.0001*	*29.96*	*<0.0001*

In a model with the concentrations of all five types of WBC included as independent variables, only heterophils significantly affected the mean luminescence of whole blood (estimate = 109 828, *F*_1,102_ = 40.97, *p* < 0.0001). Unsurprisingly, blood with higher concentrations of phagocytic heterophils exhibited higher levels of phagocytosis. Significance levels of the other four blood cell types in the model were all above *p* = 0.13.

## 4. Discussion

Although total numbers of both RBCs and WBCs were unaffected by mass change, the composition of the white cell population was substantially altered. Toads that lost mass rapidly had higher levels of heterophils and monocytes, but lower levels of eosinophils, compared with toads that gained mass. When glucocorticoids such as CORT are released, heterophils are moved into circulation while lymphocytes move out of circulation. In the present study, we observed the former pattern but not the latter. That is, heterophils increased among toads that lost mass while lymphocyte numbers remained constant. The increase in heterophil numbers alone was enough to cause a significant increase in H : L ratio. Only one of the two cell types expected to reflect stress was affected by mass loss, suggesting that the stress was not severe. Stress can also induce a reduction in eosinophils [29], as we observed among toads that lost mass.

Toads that gained more mass also had higher levels of eosinophils in circulation, suggesting that the production of these cells may impose too high an energy cost for individuals in poor energy balance. Toads in negative energy balance may invest in more economical defences such as heterophils, which enhance a general immune response (phagocytosis). Although the roles of eosinophils in ectotherms are not fully understood [43], they may enhance tissue remodelling [44]. Thus, the higher levels of eosinophils seen in individuals that gained mass may facilitate increased structural growth.

Because toads that lost mass had higher levels of heterophils and monocytes (both of which are phagocytic), they demonstrated superior performance in the phagocytosis assay. Performance in the other assays, which reflect different components of the immune system, were unaffected by body mass changes. Thus, we found no evidence to support the prediction that weight loss would weaken immune performance. Components of the immune system may be affected differentially when an organism experiences reduced energy availability [18]. Future studies could assay additional components of the immune system to look for other relationships.

We observed decreases in body mass over two to three months in 37% of captive cane toads. Major changes in body mass likely reflect foraging success and nutrition. Some minor changes in mass were undoubtedly influenced by differences in gut and bladder fullness at the time of weighing; however, this source of error should be as likely to increase as to decrease mass. Large decreases in mass among wild female toads can indicate oviposition [45], but this was not the case among captive toads, as no breeding was observed in enclosures. The fluctuations in body mass we observed in toads are likely to be largely a result of feeding rates, in turn driven by prey availability, and competition within enclosures.

Why did some captive toads in this study lose mass while others gained? This is an important issue for interpreting our immunological results because it can clarify the direction of causation for the link between changes in mass and in immune responses. That is, either a pathogen might activate immune responses that subsequently cause weight loss; or weight changes may arise through variation in food intake, and subsequently cause reconfiguration of immune cell populations. The latter scenario is more likely. Because all toads were reared in captivity under identical conditions, exposure to pathogens and parasites was minimized, or at least standardized [46]. All captive animals in this study appeared healthy, and without wounds. Variation among individuals in body mass trajectories likely reflect differences in feeding success. Within each enclosure, toads competed for flying insects that were attracted by overhead lights. Some individuals doubtless obtained more or larger prey than did others.

Does losing body mass act as a stressor in cane toads? We did not measure levels of corticosterone in our study so are unable to use that as an index of stress. The changes in mass we recorded occurred over periods of up to three months, and thus an accompanying stress response would presumably be chronic rather than acute. Among wild toads, CORT levels are lowest during the dry season, when fat stores and rates of feeding, mass growth and activity are also low [3], indicating that periods of low food availability may not always be stressful, especially if they are predictable and not severe [25,47]. Studies on toads have found no correlation between CORT levels and body condition [48–50], suggesting that energy balance is not usually a strong stressor. However, a threshold could potentially exist whereby a prolonged period without food, accompanied by sustained mass loss, does eventually trigger a stress response.

In the absence of a pronounced stress response or systemic immune reactions to infection or inflammation, the relative abundance of different white blood cell types may be affected by their costs of production and maintenance [22,51]. Phagocytic cells (heterophils, monocytes) offer rapid and general pathogen protection as part of the innate immune system, and are considered less costly than are cells such as lymphocytes that primarily play roles in acquired immunity [52]. An individual's relative investment into innate versus acquired immunity can be affected by age, sex, size or condition [18,22,51]. Under this scenario, cane toads that lose mass strategically reorganize immune investment rather than indiscriminately downregulating all immune functions. Thus, some components might be downregulated while others are upregulated, based on the costs and benefits of each under current energetic/nutritional conditions and pathogen pressures. For toads that lost mass, increasing the production of heterophils and monocytes while reducing numbers of eosinophils could reflect an economic rationalization that best served their present circumstances.

Regardless of the mechanisms linking immune performance to mass change in cane toads, there are important ecological implications. If immune function shifts in response to climatic or biotic circumstances, then risk of pathogen infection may shift as well. If reduced energy intake and altered immune function are coupled with other disease risk factors, such as increased conspecific density or sub-optimal climatic conditions [53,54], there may be strong seasonal patterns in health status.

Our results are preliminary in nature, and our interpretations speculate on complex underlying interactions. A more focused and diverse set of experiments could clarify the patterns that we observed. For example, future work could experimentally manipulate food intake rates to generate changes in body mass, thus removing any ambiguity about causal links between immune responses and changes in body mass. Density and competition effects could also be controlled or eliminated. It would be straightforward to also measure changes in CORT levels, and thus clarify the relationships of CORT both with mass change and with immune function. In particular, such work could answer the question as to the extent to which mass loss acts as a stressor in this system, and hence the degree to which shifts in immune function are attributable to stress responses rather than (or as well as) energy-driven prioritization of specific components of immune function in the face of limited energy availability.

## Data Availability

Additional data are available from the Dryad Digital Repository: https://doi.org/10.5061/dryad.7m0cfxpz3 [55].
